# Application of microbial organic fertilizers promotes the utilization of nutrients and restoration of microbial community structure and function in rhizosphere soils after dazomet fumigation

**DOI:** 10.3389/fmicb.2022.1122611

**Published:** 2023-01-18

**Authors:** Bin Huang, Yuxuan Chen, Zhouyang Pei, Lianqiang Jiang, Yu Zhang, Jing Wang, Jie Wang

**Affiliations:** ^1^Pest Integrated Management Key Laboratory of China Tobacco, Tobacco Research Institute of Chinese Academy of Agricultural Sciences, Qingdao, China; ^2^Xuancheng Modern Agricultural Industrial Park, Xuancheng, China; ^3^Sichuan Provincial Tobacco Company Liangshanzhou Company, Liangshanzhou, China

**Keywords:** dazomet, microbial organic fertilizer, rhizosphere soil microorganisms, community structure, functional prediction

## Abstract

**Introduction:**

Soil fumigant dazomet is a broad-spectrum nematicide and fungicide that can kill non-target microbes. Fungicides or organic fertilizers are often added after fumigation to improve the recovery of soil microbes. However, the effect of adding microbial organic fertilizers (MOF) after fumigation on the structure and function of rhizosphere soil microbial communities of crops is unclear.

**Methods:**

Therefore, we investigated the effects of adding Junweinong and Junlisu MOFs after dazomet fumigation on the structure and function of rhizosphere microbial communities and its relationship with soil properties and enzyme activities.

**Results and discussion:**

The results showed that the addition of these two MOFs after dazomet fumigation significantly reduced the rhizosphere soil available phosphorus, available potassium, organic matter content, and urease, alkaline phosphatase, and catalase activities, but increased the soil pH compared with the fumigation treatment. The application of MOFs after fumigation resulted in significant enrichment of bacteria such as *Gaiella*, *norank_f_Vicinamibacteraceae*, and *Flavisolibacter* and fungi such as *Peroneutypa*, *Olpidium*, and *Microascus* in the rhizosphere soil of the crop and increased the relative abundance of functional genes of 13 kinds of amino acids metabolism, pyruvate metabolism, TCA cycle, and pentose phosphate pathway as well as endophytic and epiphytic functional groups in the rhizosphere soil. In particular, NH_4_^+^-N, pH, and AK had the greatest effect on rhizosphere microorganisms. Overall, the addition of MOFs after fumigation promoted crop root nutrient uptake, enhanced rhizosphere soil microbial metabolism, allowed more beneficial communities to colonize the roots, and promoted soil microbiological health.

## 1. Introduction

With the improvement of agricultural modernization, intensive production, characterized by monoculture planting of crop types and high replanting index, has gradually become the mainstream agricultural production model, which has led to the frequent occurrence of soil-borne diseases, causing serious effects on crop yield and quality, and has now become an important bottleneck limiting sustainable agricultural development (Harrier and Watson, 2004; Haas and Défago, 2005; Piotr et al., 2014). Crop succession leads to continuous accumulation of pathogens in soil; for example, eggplant crop succession leads to severe occurrence of bacterial wilt (Wu et al., 2020) and blight (Bogoescu et al., 2014), watermelon crop succession leads to severe occurrence of wilt (Karki et al., 2021), and ginger crop succession can lead to severe ginger plague (Yan et al., 2021). This not only breaks the soil nutrient balance but also deteriorates the soil biological traits, which eventually results in the collapse of the soil microcosm and causes serious harm to crop yield and environmental safety (Bron et al., 2011; Roeland et al., 2012).

Pesticide spraying to control soil-borne diseases of crops only kills one or a few target pathogens, and the pathogens tend to develop resistance with an increasing number of applications (Fernando, 2006; Massi et al., 2021). Currently, pre-plant application of soil fumigants is one of the most convenient methods to control soil-borne root diseases. Li et al. (2022) found that dazomet (DZ) fumigation was 93.33 and 89.62% effective against *Fusarium* and *Phytophthora*, respectively, and the yield of strawberry was twice as high after fumigation. Cheng et al. (2021) found that 1,3-dichloropropene fumigation was effective against *Fusarium* and *Phytophthora*, and significantly increased the yield of early harvested tomato by 29.1%. Fang et al. (2022) found that chloropicrin fumigation was effective against *Fusarium solani*, with 100% inhibition of its mycelial growth at 1.2 and 2.4 μg/L, and significantly inhibited its respiration and reduced CO_2_ emissions (30 and 50%). However, the commercially available fumigants are broad-spectrum and non-targeted, and they kill soil pathogens while being toxic and inhibitory to beneficial soil microorganisms (Gwenaël and Stéphane, 2012).

The using of biocontrol agent or organic fertilizer after fumigation can eliminate the negative effects of fumigants by promoting soil microcosm recovery and improving soil texture, which has become a hot research topic in this field. The application of chicken manure after chloropicrin fumigation reduced the inhibition time of soil fungi and bacteria by the fumigant (Zhang et al., 2021), and the application of silica fertilizer after DZ fumigation increased the nutrient of soil and promoted the recovery of beneficial microorganisms (Li et al., 2022). Cheng et al. (2021) found that the application of *Trichoderma*, *Bacillus*, or potassium humate after 1,3-dichloropropene fumigation could increase the diversity of soil microbes and promote the recovery of microbial communities. The addition of biocontrol agent or organic fertilizer after fumigation promoted the recovery of soil microbial community structure and increased soil fertility, but the effect of microbial organic fertilizer (MOF) application after fumigation on the function of rhizosphere soil bacterial and fungal communities is unclear.

Microbial organic fertilizers is a type of fertilizer with both microbial fertilizer and organic fertilizer effect, which is compounded from specific functional microbes and organic materials mainly from plant and animal wastes (animal and poultry manure, crop straw, and brewing residues) as the source and by harmless treatment and maturation. Because of its complete decomposition, the mortality rate of insect eggs reaches more than 95%. At the same time, MOF functional bacteria are more likely to survive when applied to the soil compared with biocontrol agents. The MOFs Junweinong and Junlisu used in this study contain various strains of biocontrol and growth-promoting strains, and are prepared by solid co-fermentation with seaweed polysaccharides, bacterial bran, and biochar, which can effectively promote the colonization of beneficial microbes in soil. Seaweed polysaccharide is a multi-component mixture of marine origin, which has the function of water retention and moisturization, and can improve the colonization of beneficial microbes. Seaweed polysaccharides can improve the health of intestinal microbes by stimulating the growth of beneficial bacteria and inhibiting the proliferation of pathogenic bacteria, thereby rebuilding the intestinal microorganisms of mice (Zhang et al., 2018; You et al., 2020).

This study was aim to investigate the effects of Junweinong and Junlisu MOFs applied after dazomet fumigation on soil properties, enzyme activities, disease control, and tobacco yield, and analyze the structure and function of soil fungal and bacterial communities, as well as the main factors affecting soil microbial communities. The results of this study are important for addressing the recovery or reestablishment of rhizosphere soil non-target microbes after fumigation and promoting soil microbial health.

## 2. Materials and methods

### 2.1. Field experiment design

In July 2021, the test of dazomet fumigation and MOFs application was performed in a tobacco field in Weng’an, Guizhou (27.078472° N, 107.471555° E). The soil was first rototilled to make the soil particles fine and uniform. Subsequently, the recommended dose of dazomet mixed with soil was evenly spread on the field, and the soil was rototilled and watered to raise the soil moisture content to 40%, and then immediately covered with a polyethylene sheet. After 20 days of fumigation, the polyethylene sheet was removed for open air. After 10 days of open air, the tobacco fields were tilled, and Junweinong and Junlisu MOFs were applied to the fumigated soil at the recommended doses (1200 kg hm^–1^), followed by transplanting of tobacco seedlings. There were four treatments: (1) CK: control or no treatment; (2) DZ: fumigation with 300 kg hm^–1^ dazomet only; (3) DZF1: 300 kg hm^–1^ dazomet fumigation followed by 1,200 kg hm^–1^ Junweinong MOF; and (4) DZF2: 300 kg hm^–1^ dazomet fumigation followed by 1,200 kg hm^–1^ Junlisu MOF. These four treatments were set up as randomized group trials. Each treatment was replicated three times, and each treatment plot was 200 m^2^.

### 2.2. Soil sample collection

After 2 months of treatment with organic fertilizer application, each treatment area was sampled by the five-point sampling method, and three soil samples were selected near each sample point. The soil surface was first swept to remove debris such as leaves and gravel, and then soil samples were dug at 5–20 cm using a sterile shovel. A total of 45 sample points were taken for each treatment: every nine were combined, with five replicates of soil samples for each treatment. The resulting soil samples were removed gravel and plant residues and divided into two parts, one stored at −80°C for soil microbiological analysis, and the other air-dried for analysis of soil properties and enzyme activity detection.

### 2.3. Determination of soil physicochemical properties

Soil ammonium and nitrate nitrogen were extracted by KCl solution. Ammonium nitrogen can react with phenol to form blue indophenol dye, and nitrate nitrogen can react with *N*(1-naphthyl)-ethylenediamine hydrochloride to form red dye, both of which have maximum absorption at 630 and 543 nm, respectively (Page, 1983). Soil available phosphorus (AP) was extracted using sodium bicarbonate leachate and measured by the air fragment continuous flow analysis technique (Olsen, 1954). Soil available potassium (AK) was extracted with a neutral 1 mol/L ammonium acetate solution and determined using a flame photometer (Model 410 Flame Photometer, Sherwood Scientific Ltd., Cambridge, UK). Soil organic matter (SOM) content was determined using the potassium dichromate oxidation-volume method (Franz et al., 1996). Soil pH was measured using a precision pure water pH meter (Shanghai San-Xin Instruments Co., Ltd., Shanghai, China). Electrical conductivity (EC) was measured with an MP513 conductivity meter (Shanghai San-Xin Instruments Co., Ltd., Shanghai, China) using a 1:2.5 soil/water suspension. Soil water content was measured using the dry weight difference method.

### 2.4. Determination of soil enzyme activities

Soil alkaline phosphatase (ALP) activity was determined by the colorimetric method using the soil ALP activity assay kit and phenylphosphonic acid disodium salt dihydrate (Liang et al., 2017). Soil catalase (CAT) activity was determined by the ultraviolet (UV) absorption method using the CAT activity assay kit (Geng et al., 2017). Soil urease (UE) activity was determined by the Berthelot colorimetric method using the UE activity assay kit (Singh and Kumar, 2008). The absorbance values of ALP, CAT, and UE at 660, 240, and 630 nm, respectively, were measured using an UV-visible spectrophotometer (UV-5100B; Shanghai Yuanxi Instruments Co., Ltd., Shanghai, China) to reflect the respective enzyme activities. All kits were purchased from Beijing Box Biotechnology Co., Ltd., China.

### 2.5. Soil DNA extraction and PCR amplification

Total gDNA was extracted from each soil sample using the DNeasy PowerSoil Kit (100) Isolation Kit (QIAGEN Co., Ltd., Düsseldorf, Germany), and each sample was extracted three times and combined into one sample to ensure soil representativeness and sufficient DNA amounts for sequencing. The mass and concentration of extracted DNA were determined using 2% agarose gel electrophoresis and NanoPhotometer N50 Touch (Implen GmbH, Munich, Germany).

The V3–V4 region of the bacterial 16S rRNA gene was amplified using the universal primers 338F (5′-ACTCCTACGGGAGCAGCAG-3′) and 806R (5′-GGAC-TACHVGGGTWTCTAAT-3′) (Nan et al., 2016). To amplify the internal transcribed spacer region (ITS) of fungi, universal primers ITS1F (5′-CTTGGTCATTTAGAGGAAGTAA-3′) and ITS2R (5′-GCTGCGTTCTTCATCGATGC-3′) were used (Rachel et al., 2013). The PCR system consisted of the 5 × FastPfu buffer 4 μL, 2.5 mM dNTPs 2 μL, forward primer (5 μM) 0.8 μL, reverse primer (5 μM) 0.8 μL, FastPfu polymerase 0.4 μL, BSA 0.2 μL, template DNA 10 ng, and finally ddH_2_O was added to reach 20 μL. The procedure for the PCR reaction was as follows: initial denaturation at 95°C for 3 min, followed by 27 cycles of denaturation at 95°C for 30 s; annealing at 55°C for 30 s; extension at 72°C for 45 s; a single extension at 72°C for 10 min; and ending at 4°C. PCR products were extracted from 2% agarose gels according to the manufacturer’s instructions, purified using the AxyPrep DNA Gel Extraction Kit (Axygen Bioscience Inc., Silicon Valley, USA), and quantified using the Quantitative Analyzer Fluorometer (Promega, Madison, WI, USA).

### 2.6. Sequence processing

The original data were sequence spliced using FLASH (v.1.2.11), and the original sequences were quality controlled using Quantitative Insights into Microbial Ecology (QIIME; v.1.9.1). Chimeric sequences were detected and removed according to the UCHIME algorithm. Using Uparse (v.11), the obtained high-quality sequences were clustered with operational taxonomic units (OTUs) at a similarity threshold of ≥97% (Edgar et al., 2011). The Silva (v.138) database based on the Mothur algorithm was used for bacteria, and the Unite (v.8.0) database based on the BLAST algorithm was used for fungi. Species annotation of bacterial and fungal sequences was performed using representative sequences of bacterial and fungal OTUs, respectively (Wang et al., 2007). The OTU abundance was homogenized in the number of sequences from the sample with the least number of sequences, and subsequent diversity analyses were performed based on the standardized OTU abundance data obtained here.

### 2.7. Bioinformatics analysis

The α-diversity of fungal communities was analyzed by the Chao and Shannon indices using Mothur (v.1.30.0) software (Schloss et al., 2009). Chao indices were used to characterize microbial community richness, while Shannon indices were used to describe microbial community diversity. Redundancy analysis (RDA) was performed using the “vegan” R (v.3.3.1) package, and principal coordinate analysis (PCoA) was performed using direct mapping based on Euclidean distances. Linear discriminant analysis effect size (LEfSe) analysis (LDA ≥ 2) was performed to discriminate samples linearly according to different classification methods (Segata et al., 2011). Species with significant differences in sample classification were identified using linear discriminant analysis (LDA). The “pheatmap” and “vegan” packages were used to generate heatmaps and statistical correlations (Kolde, 2015). Statistical analyses were performed using SPSS v.25 (SPSS Inc., Chicago, USA). Changes in soil properties and enzyme activities were analyzed by analysis of variance and Duncan’s new multiple range test. Visualization plots for environmental factor correlation analysis were drawn using R software. OriginPro 2018 (OriginLab Corp., Massachusetts, USA), and Adobe Illustrator CC2019 (Adobe Systems Inc., California, USA) software were used for plotting.

## 3. Results and analysis

### 3.1. Changes in soil physicochemical properties

Compared with CK, the AP, AK, and SOM contents of each treatment were significantly reduced and differed among the DZ, DZF1, and DZF2 treatments, with the AP, AK, and SOM contents significantly higher after the DZ treatment than after DZF1 and DZF2 treatments. Soil pH of the CK treatment was significantly lower than that of the three treatments, the highest of which was the DZF1, indicating that fumigation could improve soil pH and alleviate soil acidification. Soil ammonium nitrogen content was the highest in the CK and not significantly higher than the DZF2 treatment, but both CK and DZF2 were significantly higher than the DZF1 treatment. Soil nitrate nitrogen content was the highest in the DZ treatment and significantly higher than any other treatment, the lowest of which was the DZF2. Soil EC was the highest in the CK and the lowest in the DZF1 (Table 1).

**TABLE 1:** Soil physicochemical properties for different treatments.

Treatment	NH_4_^+^-N (mg/kg)	NO_3_^–^-N (mg/kg)	Available P (mg/kg)	Available K (mg/kg)	Organic matter (mg/kg)	pH (1:2.5)	EC (μ s/cm)
CK	115.81 ± 16.87a	294.84 ± 5.30b	105.46 ± 4.59a	2010.47 ± 17.26a	68.10 ± 3.11a	5.83 ± 0.11c	966.00 ± 46.26a
DZ	66.57 ± 5.36bc	321.50 ± 8.39a	84.23 ± 5.36b	1376.80 ± 98.46b	50.17 ± 3.08b	6.12 ± 0.04ab	881.67 ± 39.01ab
DZF1	57.32 ± 6.47c	198.07 ± 7.85c	56.24 ± 4.19c	939.51 ± 54.58d	33.13 ± 1.93c	6.29 ± 0.04a	718.00 ± 48.82c
DZF2	92.91 ± 4.54ab	114.55 ± 6.35d	55.81 ± 6.94c	1156.07 ± 47.00c	29.97 ± 2.62c	6.00 ± 0.08bc	785.00 ± 23.09bc

CK, no treatment; DZ, fumigation with 300 kg hm^–1^ dazomet; DZF1, 300 kg hm^–1^ dazomet fumigation followed by 1,200 kg hm^–1^ Junweinong MOF; DZF2, 300 kg hm^–1^ dazomet fumigation followed by 1,200 kg hm^–1^ Junlisu MOF; NH_4_^+^-N, ammonium nitrogen; NO_3_^–^-N, nitrate nitrogen; EC, electrical conductance; Means (N = 3) within the same column accompanied by the same letter following by Duncan’s new multiple range test are not statistically different (*p* = 0.05. a, b, and ab are significant markers, different letters indicate significant differences, as long as one letter is the same, it is not significant).

### 3.2. Changes in soil enzyme activities

**FIGURE 1:**
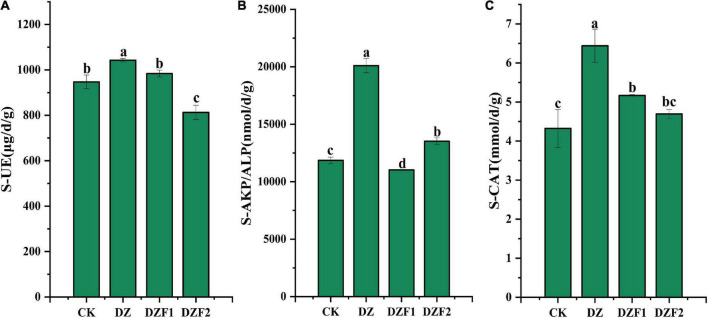
Activities of three soil enzymes after different treatments. **(A)** Soil urease activity; **(B)** Soil alkaline phosphatase activity; **(C)** Soil catalase activity. CK, no treatment; DZ, fumigation with 300 kg hm^–1^ dazomet; DZF1, 300 kg hm^–1^ dazomet fumigation followed by 1,200 kg hm^–1^ Junweinong MOF; DZF2, 300 kg hm^–1^ dazomet fumigation followed by 1,200 kg hm^–1^ Junlisu MOF. The error bars represent the standard errors.

The DZF2 treatment significantly reduced soil UE activity (14.19%) and increased soil ALP activity (14.05%) compared with the control (Figure 1). Compared with the control, the DZF1 treatment significantly increased soil CAT activity (19.57%) and significantly decreased soil ALP activity (6.97%), although it did not significantly change soil UE activity. Compared with the control, the DZ treatment significantly increased soil UE, ALP, and CAT activities (10.10, 69.57, and 48.99%), which were also significantly higher than the DZF1 and DZF2 treatments.

### 3.3. Changes in diversity of soil microorganisms

**FIGURE 2:**
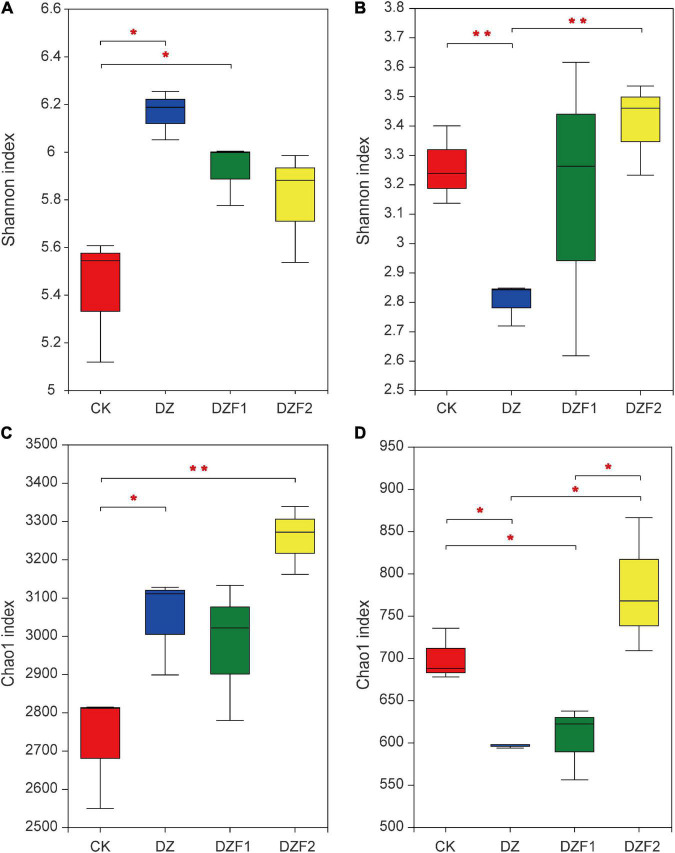
Changes in α-diversity of soil microorganisms. **(A)** Shannon index of soil bacteria; **(B)** Shannon index of soil fungi; **(C)** Chao1 index of soil bacteria; **(D)** Chao1 index of soil fungi. The error bars represent the standard errors. “*” Represents a significant difference at *p* < 0.05, “**” represents a significant difference at *p* < 0.01.

Compared with the CK, the Shannon index of the bacterial community was significantly higher in both DZ and DZF1 treatments, among which the DZ reached the highest (Figure 2A), and the Chao1 index of the bacterial community was significantly higher in both DZ and DZF2, among which the DZF2 was the highest and was also significantly higher than the DZ (Figure 2C), indicating that Junlisu MOF could contribute to the increase in bacterial diversity after fumigation. The DZ had the lowest Shannon and Chao1 indices (Figures 2B, D), indicating that fumigation reduced the diversity and abundance of soil fungi. Compared with fumigation alone, DZF2 significantly increased the Shannon and Chao1 indices of the fungal community, indicating that the application of Junlisu MOF after fumigation helped to promote the diversity and abundance of soil fungi after fumigation.

**FIGURE 3:**
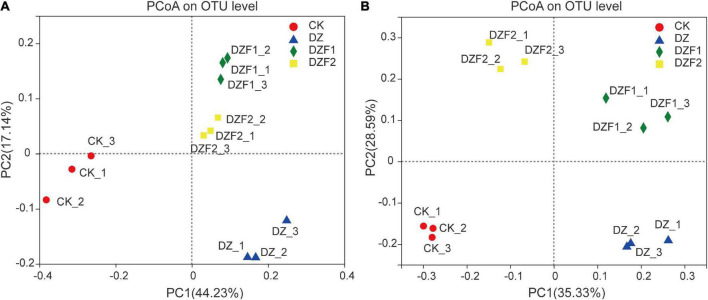
Changes in β-diversity of soil microorganisms. **(A)** Principal coordinate analysis (PCoA) of soil bacteria; **(B)** PCoA of soil fungi.

The PCoA results showed that treatments with MOFs (DZF1 and DZF2) applied after fumigation, fumigation alone (DZ), and CK were distributed in different quadrants, and the different treatments were separated by PC1 and PC2 axes. The contribution of PC1 and PC2 to the difference in microbial composition between treatments was 44.23/17.14% (bacteria) and 35.33/28.59% (fungi) (Figures 3A, B). The treatments for bacteria and fungi could be clearly separated from CK by the PC1 axis, and although DZF1 and DZF2 were close to DZ on the PC1 axis, they could be clearly separated by the PC2 axis, indicating that the application of Junweinong and Junlisu MOFs after dazomet fumigation could change the microbial community affected by dazomet fumigation.

### 3.4. Analysis of soil microbial composition

**FIGURE 4:**
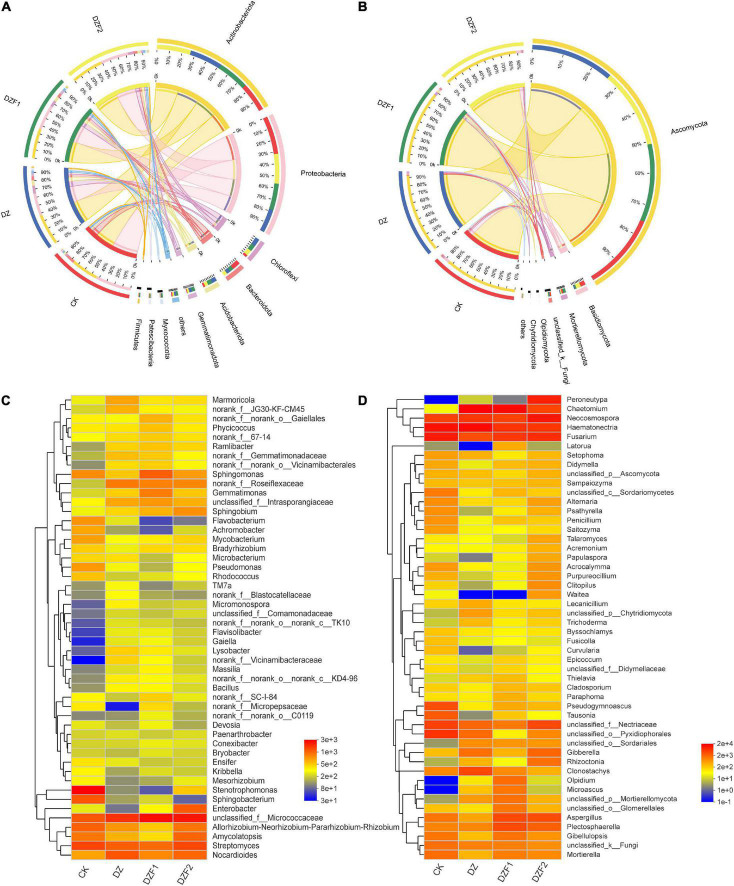
Analysis of soil microbial composition. **(A)** Circos analysis of soil bacteria; **(B)** Circos analysis of soil fungi; **(C)** Heatmap of the soil bacterial community; **(D)** Heatmap of the soil fungal community.

The Cricos plot results showed that the composition of the dominant species in the different samples was similar. In the CK, DZ, DZF1, and DZF2 treatments, the dominant bacterial phyla were Actinobacteriota (22.67, 26.01, 24.62, and 26.70%) and Proteobacteria (32.36, 20.06, 22.40, and 25.19%), followed by Chloroflexi (10.71, 35.47, 28.47, and 25.34%), Bacteroidota (41.97, 19.87, 20.39, and 17.77%), Acidobacteriota (13.17, 33.15, 31.11, and 22.57%). The dominant fungal phylum was Ascomycota (24.25, 26.20, 24.51, and 25.04%), followed by Basidiomycota (43.18, 7.91, 11.58, and 37.33%), Mortierellomycota (25.02, 5.11, 45.30, and 16.51%), unclassified_k_Fungi (36.05, 3.93, 26.33, and 16.22%), and Olpidiomycota (0.20, 5.22, 93.46, and 1.11%) (Figures 4A, B).

Figures 4C, D shows the changes in soil microorganisms at the genus level for all treatments. The top five soil bacteria at the genus level were *unclassified_f_Micrococcaceae*, *Streptomycs*, *Nocardiodes*, *Stenotrophomonas*, and *Allorhizobium-Neorhizobium-Parararhizobium-Rhizobium*, and the fumigation treatment increased the relative abundance of *Gaiella*, *norank_f_Vicinamibacteraceae*, and *Flavisolibacter* and decreased the relative abundance of *Stenotrophomonas* compared with CK (Figures 4A, C). The top five soil fungi at the genus level were *Haematonectria*, *Chaetomium*, *Fusarium*, *Neocosmospora*, and *unclassified_f_Nectriaceae*, and the fumigation treatment increased the relative abundance of *Peroneutypa*, *Olpidium*, and *Microascus* compared with CK (Figures 4B, D).

### 3.5. Differences in soil microbial community at genus level in different treatments

**FIGURE 5:**
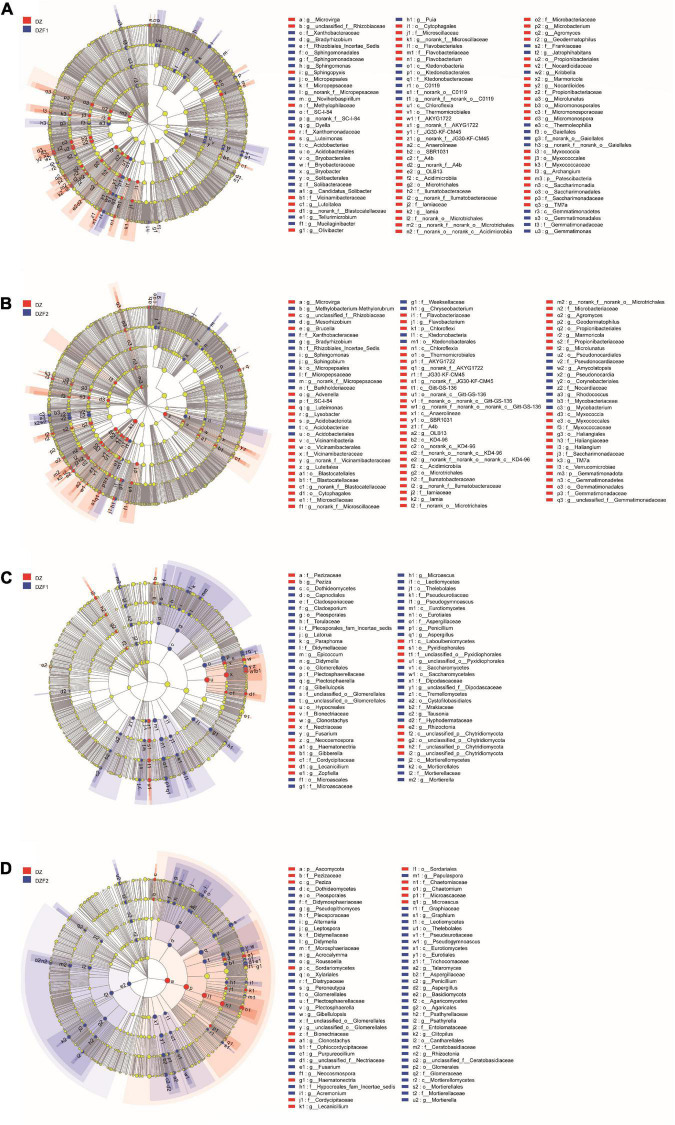
Linear discriminant analysis effect size (LEfSe) of soil microorganisms. **(A)** LEfSe of soil bacteria between DZ and DZF1; **(B)** LEfSe of soil bacteria between DZ and DZF2; **(C)** LEfSe of soil fungi between DZ and DZF1; **(D)** LEfSe of soil fungi between DZ and DZF2.

Figure 5 shows the differences in microbial communities from the phylum to genus level between different MOFs applied after fumigation and fumigation alone. In the soil bacterial community, Patescibacteria was enriched in the DZ compared with the DZF1 treatment, while Sphingomonadales, Micropepsales, Gaiellales, Gemmatimonadales, C0119, Solibacterales, Acidobacteriales, Ktedonobacterales, and Bryobacterales were significantly enriched in the DZF1 treatment at the class level, and *Sphingomonas*, *Gemmatimonas*, *Bradyrhizobium*, *Mucilaginibacter*, *Jatrophihabitans*, *Noviherbaspirillum*, *Tellurimicrobium*, *Dyella*, *Kribbella*, and *Bryobacter* were significantly enriched in the DZF1 treatment at the genus level (Figure 5A). Compared with DZF2, Chloroflexi, Acidobacteria, and Gemmatimonadota were enriched in DZ, while Pseudonocardiales, Corynebacteriales, Micropepsales, Ktedonobacterales, and Acidobacteriales were significantly enriched in the DZF2 treatment at the class level. *Amycolatopsis*, *Sphingobium*, *Chryseobacterium*, *Sphingomonas*, *Bradyrhizobium*, *Mycobacterium*, *Mesorhizobium*, *Rhodococcus*, *Pseudonocardia*, and *Methylobacterium-Methylorubrum* were significantly enriched in the DZF2 treatment at the genus level (Figure 5B). In the soil fungal community, Hypocreales and Pyxidiophorales were enriched in the DZ treatment compared with the DZF1 treatment. Glomerellales, Eurotiales, Microascales, Pleosporales, Mortierellales, Thelebolales, Saccharomycetales, Capnodiales, and Cystofilobasidiales were significantly enriched in the DZF1 treatment at the class level compared with the DZ treatment, and *Aspergillus*, *Fusarium*, *Plectosphaerella*, *Gibellulopsis*, *Microascus*, *Mortierella*, *Latorua*, *Pseudogymnoascus*, *Paraphoma*, *Didymella*, *Cladosporium*, *Tausonia*, *Penicillium*, and *Epicoccum* were significantly enriched in the DZF1 treatment at the genus level (Figure 5C). Ascomycota significantly enriched in DZ treatment and Basidiomycota significantly enriched in DZF2, and Xylariales, Eurotiales, Cantharellales, Glomerellales, Pleosporales, Agaricales, Mortierellales, and Thelebolales were significantly enriched in the DZF2 treatment at the class level (Figure 5D).

### 3.6. Correlation analysis of soil properties and enzyme activities with microorganisms

**FIGURE 6:**
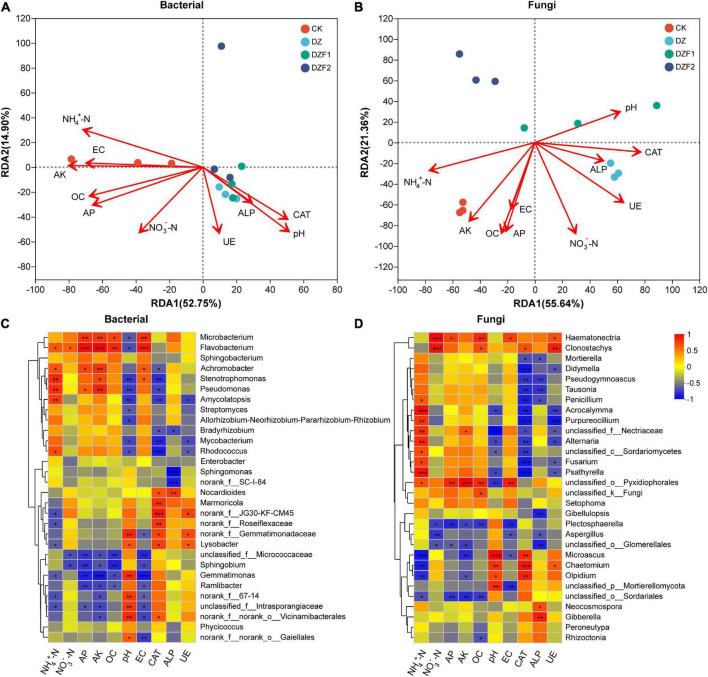
Correlation analysis of soil physicochemical properties and enzyme activities with microorganisms. **(A)** Redundancy analysis (RDA) of soil physicochemical properties and enzyme activities with soil bacteria; **(B)** RDA of soil physicochemical properties and enzyme activities with soil fungi; **(C)** Heatmap analysis of correlation of soil physicochemical properties and enzyme activities with soil bacteria; **(D)** Heatmap analysis of correlation of soil physicochemical properties and enzyme activities with soil fungi. ALP, alkaline phosphatase; UE, urease; CAT, catalase; NH_4_^+^-N, ammonium nitrogen; NO_3_^–^-N, nitrate nitrogen; EC, electrical conductance. “*” Represents a significant difference at *p* < 0.05; “**” represents a significant difference at *p* < 0.01; “***” represents a significant difference at *p* < 0.001.

We analyzed the correlation between soil physicochemical properties and enzyme activities with microbial communities by RDA. 52.75/14.90% and 55.64/21.36% were explained by RDA1 and RDA2 axes for soil bacteria and fungi, respectively (Figures 6A, B). In the soil bacterial community, the most and least affected were AK and ALP, respectively. In the soil fungal community, the most and least affected were NH_4_^+^-N and ALP, respectively. Overall, there was a positive correlation between NH_4_^+^-N, EZ, AK, OC, and AP, and a positive correlation between pH, ALP, CAT, and UE for both soil bacteria and soil fungi, but there was a negative correlation between these two treatments. The soil bacterial community was positively correlated with the CK and negatively correlated with the DZ, DZF1, and DZF2, while the soil fungal community was just the opposite. Therefore, MOF application after fumigation could change soil pH, thereby increasing soil enzyme activities and improving the soil.

Correlation heatmaps were used to assess correlations between soil physicochemical properties and enzyme activities with microorganisms at the genus level (Figures 6C, D). AK showed significant positive correlations with *Microbacterium*, *Flavobacterium*, *Achromobacter*, *Stenotrophomonas*, *Pseudomonas*, *Nectriaceae*, and *Pyxidiophorales* and negative correlations with *Gemmatimonas*, *Microascus*, and *Sordariales*. pH showed significant positive correlations with *Gemmatimonadaceae*, *Lysobacter*, *Gemmatimonas*, *Intrasporangiaceae*, *Vicinamibacterales*, *Gaiellales*, *Microascus*, *Chaetomium*, *Olpidium*, and *Mortierellomycota* and negative correlations with *Microbacterium*, *Flavobacterium*, *Stenotrophomonas*, *Pseudomonas*, *Amycolatopsis*, *Streptomyces*, *Allorhizobium-Neorhizobium-Pararhizobium-Rhizobium*, *Mycobacterium*, *Rhodococcus*, *Acrocalymma*, *Nectriaceae*, *Alternaria*, *Psathyrella*, and *Pyxidiophorales*. On the whole, the correlations of pH, ALP, CAT, and UE with the same soil microorganisms had similarities, unlike other soil physicochemical properties, which were also consistent with the RDA results.

### 3.7. Prediction analysis of functional genes of microbial metabolic pathways

**FIGURE 7:**
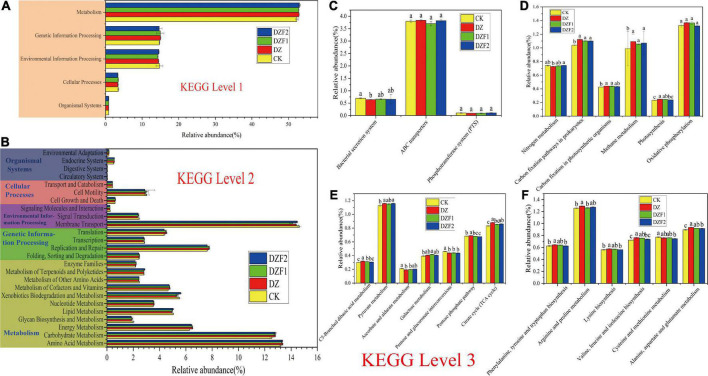
Prediction analysis of functional genes of soil bacterial metabolic pathways. **(A)** Prediction of soil bacterial metabolic pathways at level 1; **(B)** Prediction of soil bacterial metabolic pathways at level 2; **(C)** Prediction of functions at level 3 of membrane transport; **(D)** Prediction of functions at level 3 of energy metabolism; **(E)** Prediction of functions at level 3 of carbohydrate metabolism; **(F)** Prediction of functions at level 3 of amino acid metabolism.

The functions of soil bacteria were studied using the KEGG database. KEGG functions are classified into six major categories at level 1, of which we studied the top five functions, namely: metabolism (52.26–52.99%), genetic information processing (14.67–15.01%), environmental information processing (14.28–14.74%), cellular processes (3.36–3.48%), and organismal systems (0.84–0.86%) (Figure 7A). The main pathways of KEGG at level 2 in this study were membrane transport (13.94–14.46%), amino acid metabolism (13.05–13.20%), carbohydrate metabolism (12.38–12.65%), replication and repair (7.53–7.68%), and energy metabolism (6.20–6.44%) (Figure 7B). Compared with the control, the correlation abundance of functional genes was significantly higher in each treatment for energy metabolism, amino acid metabolism, carbohydrate metabolism, and membrane transport, as the functional genes with the greatest correlation abundance at level 2 had significantly lower correlation abundance in each treatment than the control.

The abundance of ABC Transports functional genes was the highest in membrane transport, but no significant difference was observed between its four treatments (Figure 7C). The DZ treatment reduced the relative abundance of bacterial secretion system functional genes compared with CK, but this effect was improved by DZF1 and DZF2 treatments and restored to CK levels. In energy metabolism (Figure 7D), the top five metabolic pathways were oxidative phosphorylation, carbon fixation pathways in prokaryotes, methane metabolism, nitrogen metabolism, and carbon fixation in photosynthetic organisms. Among these, oxidative phosphorylation did not differ significantly among all treatments. The abundance of functional genes associated with carbon fixation pathways in prokaryotes, methane metabolism, carbon fixation in photosynthetic organisms and photosynthesis was significantly higher compared with the control. Among the nitrogen metabolic pathways, the lowest abundance was found in the DZ treatment, and the abundance in the DZF1 and DZF2 treatments was significantly higher than that in the DZ treatment and not significantly different from that in the CK treatment. Thus, nitrogen metabolism was impaired after fumigation, and MOF application could promote the recovery of nitrogen metabolism after fumigation. In carbohydrate metabolism (Figure 7E), the top five metabolic pathways were butanoate metabolism, propionate metabolism, pyruvate metabolism, glycolysis/gluconeogenesis, and citrate cycle (TCA cycle). Compared with the control, the DZ treatment significantly increased the abundance of genes associated with C5-branched dibasic acid metabolism, pyruvate metabolism, pentose phosphate pathway, and citrate cycle functions, while significantly decreased the abundance of genes associated with ascorbate and aldarate metabolism, and pentose and glucuronide interconversion functions. Compared with the DZ treatment, the DZF1 and DZF2 treatments showed significantly reduced abundance of functional genes in the C5-branched dibasic acid metabolism and pentose phosphate pathways. The top five amino acid metabolic pathways were arginine and proline metabolism; valine, leucine, and isoleucine degradation; glycine, serine, and threonine metabolism; alanine, aspartate, and glutamate metabolism; and tryptophan metabolism (Figure 7F). The DZ treatment significantly increased the relative abundance of functional genes for phenylalanine, tyrosine, and tryptophan biosynthesis; arginine and proline metabolism; lysine biosynthesis; valine, leucine, and isoleucine biosynthesis; and alanine, aspartate, and glutamate metabolism compared with CK. Compared with the DZ treatment, the DZF1 treatment significantly reduced the relative abundance of functional genes for arginine and proline metabolism, and the DZF2 treatment significantly reduced the relative abundance of functional genes for phenylalanine, tyrosine, and tryptophan biosynthesis; lysine biosynthesis; valine, leucine, and isoleucine biosynthesis; cysteine and methionine metabolism; and alanine, aspartate, and glutamate metabolism. Among these metabolic pathways, the relative abundance of functional genes for cysteine and methionine metabolism was the highest in the CK treatment.

**FIGURE 8:**
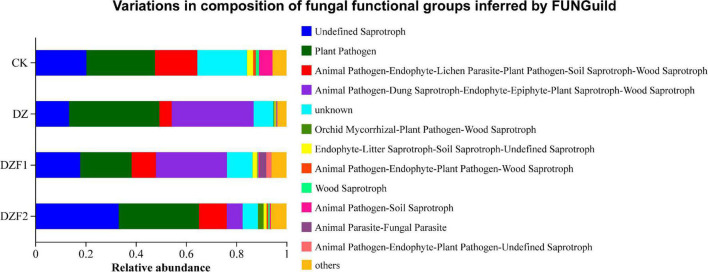
Prediction analysis of functional genes of soil fungal metabolic pathways.

The functions of soil fungi were predicted using FUNGuild (Figure 8). Among these, undefined saprotroph, plant pathogen, animal pathogens-endophyte-lichen parasite-plant pathogen-soil saprotroph-wood saprotroph, animal pathogen-dung saprotroph-endophyte-epiphyte-plant saprotroph-wood saprotroph were the main functional members of the fungal community, with saprotrophs and plant pathogens constituting the majority (50%). The abundance of undefined saprotroph was reduced in the DZ treatment compared with the CK treatment, and this phenomenon was alleviated in the DZF1 and DZF2 treatments, and the abundance of undefined saprotroph was significantly higher in the DZF2 treatment than in the CK treatment, showing that DZF2 had a better effect on the reduction in saprotroph abundance caused by dazomet fumigation. Notably, the category of animal pathogen-dung saprotroph-endophyte-epiphyte-plant saprotroph-wood saprotroph with saprophytic functions was almost absent in the CK treatment, but the abundance was significantly higher in the DZ, DZF1, and DZF2 treatments, indicating that fumigation increased the abundance of most saprophytic bacteria, which was helpful for the restoration of soil functions.

## 4. Discussion

### 4.1. Effect of adding MOFs after dazomet fumigation on soil physicochemical properties

Fumigation and post-fumigation application of organic fertilizer can increase soil pH, which is consistent with the findings of Cheng et al. (2021). Soil pH is a key factor affecting soil health, and black shank and black root rot of tobacco have high incidence at pH 4.8–5.8. Rhizoctonia and sclerotinia diseases of cruciferous plants are prone to occur in acidic soils and hardly occur at pH 7.2–7.4 after changing soil acidity. Most plant pathogens are acidic soil lovers, therefore, increasing soil pH is effective in protecting plants against plant pathogens. In addition, the content of AP, AK, and SOM was significantly reduced in each treatment due to the presence of potassium humate in the MOF we used. Potassium humate, a bioactive agent, strongly stimulates microbial metabolism and effectively promotes potassium uptake and utilization by plants, both of which increase nutrient consumption and reduce the content of AP, AK, and SOM (Swify et al., 2022).

### 4.2. Effect of adding MOFs after dazomet fumigation on soil enzyme activities

The application of organic fertilizer after fumigation can increase the activity of soil peroxidase and sucrase (Li et al., 2022). This is undeniable because organic fertilizers can improve soil structure, insulate and moisturize the soil, decompose organic matter, provide trace elements, etc. However, our results are contrary to the results obtained by Li et al. (2021a), because the soil we collected was rhizosphere soil rather than non-rhizosphere soil. Root exudates of saline plants have a chemotactic, biofilm forming, and colonizing effect on the thermophilic bacterium *Halomonas anticariensis* FP35T (Inmaculada et al., 2020). We hypothesize that tobacco root secretions recruit beneficial microorganisms from microbial fertilizers, resulting in high microbial metabolism in the root base region, depletion of soil nutrients and vitality, and reduced enzyme activities.

### 4.3. Effect of adding MOFs after dazomet fumigation on soil microbial diversity

For rhizosphere soil bacteria, both fumigation and post-fumigation addition of MOF increased their Shannon and Chao1 indices. For soil fungi, dazomet fumigation decreased the Shannon and Chao1 indices, which is consistent with the results of Zhu et al. (2020). This is because of the lack of selectivity of fumigants on the inhibitory effect of soil microorganisms. Meanwhile, the Shannon and Chao1 indices of soil fungi were significantly improved after MOF application, which is because MOF contains sufficient nutrients and various beneficial microorganisms that stimulate the growth and reproduction of soil microorganisms and increase the diversity of microbial species in the region. The β-diversity of bacterial and fungal communities in each treatment was significantly different from the control, and the difference between MOF application after fumigation and fumigation alone was also significant. Combined with the α-diversity, we can conclude that the addition of MOF after fumigation significantly changed the community composition of soil microorganisms.

### 4.4. Effect of adding MOFs after dazomet fumigation on soil microbial composition

Among soil bacteria, the Actinobacteriota and Proteobacteria are the most abundant in soil. Actinobacteriota can promote the decay of plant and animal remains in soil. The use of streptomycin can change the structure of soil microbial communities, reduce the number of harmful soil microorganisms, and exhibit various beneficial functions (Carlos et al., 2013). Proteobacteria is also important in soil ecological safety, and they are involved in the soil nitrogen cycle. The highest abundance of soil fungi is Ascomycota. Eleonora Egidi collected soil samples from 18 countries on six continents and found that Ascomycota is the most dominant species in soils worldwide, and they contribute significantly to soil ecological safety as an important component of soil microbial communities and as pioneer species among decomposers (Eleonora et al., 2019).

Fumigation treatments increased the relative abundance of *Gaiella*, *norank_f_Vicinamibacteraceae*, and *Flavisolibacter* compared with the control. *Gaiella*-dominated microbial communities can increase systemic resistance and thus suppress Mongolian *Astragalus membranaceus* root rot (Li et al., 2021b). After continuous application of isooxadiazon fumigation, a bacterial community such as *Vicinamibacteraceae*, which can degrade complex carbon compounds and bioremediation, became a new community module hub (Du et al., 2018a). *Flavisolibacter*, as an inter-rhizosphere probiotic, can colonize roots more effectively than other soil microorganisms, and they are able to limit the growth of *Fusarium oxysporum* (Sohail et al., 2018).

Linear discriminant analysis effect size showed differences between fumigation followed by MOF application and fumigation alone. Our results indicate that fumigation followed by MOF application can increase the relative abundance of beneficial and functional microbial communities in soil. Zhang identified a degrading strain SHPJ-2 belonging to *Sphingomonadales* that can degrade polycyclic and heterocyclic aromatic hydrocarbons and promote the recovery of soil contaminated with toxic organic matter (Zhang et al., 2022). *Solibacterales* are important for phosphorus bioaccumulation, solubilization of inorganic phosphorus, and mineralization of organic phosphorus. And as an important component of cellular protoplasm, *Solibacterales* is very closely related to cell division during crop growth. For significantly different soil fungi, *Mortierella*, a phosphorus solubilizing fungus, is indispensable for soil carbon and nitrogen cycling (Osorio and Mitiku, 2014).

### 4.5. Correlation analysis of soil physicochemical properties and enzyme activities with microorganisms

A significant correlation was found between soil physicochemical properties and enzyme activities. In soil bacteria, there was a significant positive correlation between pH and activities of three soil enzymes, which is consistent with previous results (Cheng et al., 2020). Our results clearly divide these factors into two categories: (1) NH_4_^+^-N, EC, AK, OC, and AP, and (2) pH, ALP, CAT, and UE, and the correlations between them and soil microorganisms were similar. Our results showed that all treatments increased soil pH. The bacterial group significantly correlated with pH among soil bacteria was *Bacillus*, and *Bacillus aurantiaca* plays an important role in N_2_O reduction in agricultural soils (Oshiki et al., 2022). The fungal group most related to soil pH is *Microascus*, which is a biologically active microorganism with antibacterial and antitumor activities (National Microbial Rescoure Center, 2013).

### 4.6. Prediction analysis of functional genes of microbial metabolic pathways

For the functional prediction of soil bacteria, we focused on metabolism, which accounts for more than half of the metabolism at level 1. The abundance of functional genes for energy metabolism, carbohydrate metabolism, and amino acid metabolism was significantly higher in the top three abundances at level 2 for each treatment compared with the control, which is consistent with the findings of Huang et al. (2020), thus, we continued to investigate the specific metabolic pathways at level 3 for these three metabolic pathways. In energy metabolism, dazomet fumigation reduced the relative abundance of functional genes for nitrogen metabolism, but the addition of MOFs after fumigation improved this situation. Previous studies have shown that all five fumigants reduced the number of denitrifying bacteria containing genes encoding napA, narG, nirS, or nirK enzymes and reduced the intensity of nitrogen metabolism by soil bacteria (Fang et al., 2020). In amino acid metabolism, fumigation increased the abundance of functional genes for the metabolism of 13 amino acids and only decreased the abundance of functional genes for the metabolism of cysteine and methionine. Soon after fumigation, microorganisms die and cells lyse, which produce activated carbon and phospholipids, which in turn stimulate the amino acid metabolism of living microorganisms and perform various biochemical reactions to complete microbial life activities (Huang et al., 2020). Carbohydrates are important SOM components, which not only indicate changes in SOM and soil microorganisms but also stabilize soil structure and maintain the stability of the soil environment. In carbohydrate metabolism, pyruvate connects the three major metabolism of glucose, fatty acids, and amino acids through acetyl CoA. Our results showed that pyruvate metabolism was enhanced after fumigation, whereas the TCA cycle and pentose phosphate pathway were enhanced to promote glucose decomposition and soil metabolism was vigorous, which is a proof of soil ecological recovery.

For the functional prediction of soil fungi, we found that the metabolism of saprophytic fungi in soil was significantly enhanced after fumigation. The presence of a large number of saprophytic fungi enhances the ability of the soil to decompose organic matter and dead host cells, thus enhancing soil nutrient cycling, which has an improved effect on plant growth (Du et al., 2018b). In addition, the massive enhancement of metabolism of endophytic and epiphytic fungi is effective in plant disease control. Plant endophytic bacteria have the same ecological niche as pathogenic bacteria and compete for space and nutrients in the plant; thus, pathogenic bacteria do not receive normal nutrient supply and die out, thereby enhancing host resistance (Adeleke et al., 2022). Furthermore, plant endophytes can secrete metabolites such as antibiotics and toxins, which can induce systemic resistance in plants.

## 5. Conclusion

The addition of Junweinong and Junlisu MOFs after dazomet fumigation significantly reduced the AP, AK, and SOM contents of inter-root soil, whereas UE, ALP, and CAT activities were reduced to varying degrees and increased soil pH, all of which are a sign of vigorous inter-root soil microbial metabolism. High soil pH is detrimental to the survival of soil pathogenic bacteria and contributes to plant health. The addition of both MOFs after fumigation increased the abundance of rhizosphere bacteria without significant effect on microbial diversity. However, it changed the composition of rhizosphere microorganisms, as bacteria such as *Gaiella*, *norank_f_Vicinamibacteraceae*, and *Flavisolibacter* and fungi such as *Peroneutypa*, *Olpidium*, and *Microascus* were significantly enriched. These microorganisms have important roles in enhancing crop system resistance and promoting nutrient uptake. In addition, the addition of MOF after dazomet fumigation increased the relative abundance of functional genes for 13 kinds of amino acid metabolism, pyruvate metabolism, TCA cycle, and pentose phosphate pathway in the rhizosphere soil. Further, the significant increase in the relative abundance of saprophytic bacteria as well as endophytic and epiphytic functional groups may increase the decomposition of SOM and resist pathogen invasion. In conclusion, the addition of MOF after fumigation can enhance rhizosphere soil microbial metabolism, promote crop root nutrient uptake, improve soil acidification, and promote the recovery of soil microbial communities, allowing more beneficial bacteria to colonize, with positive effects on soil microbial health.

## Data availability statement

The sequences were deposited in the NCBI Sequence Read Archive (SRA) database (Accession number: SRP414818).

## Author contributions

JieW: conceptualization. YC and BH: methodology, software, and writing—original draft preparation. YC and JieW: validation. ZP, YZ, JinW, and LJ: investigation. BH: writing—review and editing. ZP and JieW: supervision. JieW and BH: funding acquisition. All authors have read and agreed to the published version of the manuscript.

## References

[B1] Adeleke B. S. Fadiji A. E. Ayilara M. S. Igiehon O. N. Nwachukwu B. C. Babalola O. O. (2022). Strategies to enhance the use of endophytes as bioinoculants in agriculture. *Horticulturae* 8:498. 10.3390/HORTICULTURAE8060498

[B2] Bogoescu M. Doltu M. Sora D. Be Gullino M. L. Pugliese M. Katan J. (2014). Prevention and control of soilborne diseases and nematodes in eggplants crop by grafting plants combined with soil fumigation. *Acta Hortic. Sin.* 1044 331–336. 10.17660/ActaHortic.2014.1044.43

[B3] Bron P. A. van Baarlen P. Kleerebezem M. (2011). Emerging molecular insights into the interaction between probiotics and the host intestinal mucosa. *Nat. Rev. Microbiol.* 10 66–78. 10.1038/nrmicro2690 22101918

[B4] Carlos R. B. Luis A. A. Diego A. A. Héctor C. A. Eddie S. (2013). Evaluation of pesticide residues in open field and greenhouse tomatoes from Colombia. *Food Control* 30 400–403. 10.1016/j.foodcont.2012.08.015

[B5] Cheng H. Zhang D. Huang B. Song Z. Ren L. Hao B. (2020). Organic fertilizer improves soil fertility and restores the bacterial community after 1,3-dichloropropene fumigation. *Sci. Total Environ.* 738 PP140345–140345. 10.1016/j.scitotenv.2020.140345 32806339

[B6] Cheng H. Zhang D. Ren L. Song Z. Li Q. Wu J. (2021). Bio-activation of soil with beneficial microbes after soil fumigation reduces soil-borne pathogens and increases tomato yield. *Environ. Pollut.* 283:117600. 10.1016/J.ENVPOL.2021.117160 33878684

[B7] Du P. Wu X. Xu J. Dong F. Liu X. Zheng Y. (2018a). Effects of trifluralin on the soil microbial community and functional groups involved in nitrogen cycling. *J. Hazard. Mater.* 353 204–213. 10.1016/j.jhazmat.2018.04.012 29674095

[B8] Du P. Wu X. Xu J. Dong F. Liu X. Zhang Y. (2018b). Clomazone influence soil microbial community and soil nitrogen cycling. *Sci. Total Environ.* 644 475–485. 10.1016/j.scitotenv.2018.06.214 29990898

[B9] Edgar R. C. Haas B. J. Clemente J. C. Quince C. Knight R. (2011). UCHIME improves sensitivity and speed of chimera detection. *Bioinformatics* 27 2194–2200. 10.1093/bioinformatics/btr381 21700674 PMC3150044

[B10] Eleonora E. Manuel D. Jonathan M. P. Wang J. David J. E. Richard D. B. (2019). A few Ascomycota taxa dominate soil fungal communities worldwide. *Nat. Commun.* 10:2369. 10.1038/s41467-019-10373-z 31147554 PMC6542806

[B11] Fang W. Liu X. Song Z. Jin X. Yan D. Wang Q. (2022). Mechanism of the antifungal action of chloropicrin fumigation against *Panax notoginseng* root rot caused by *Fusarium solani*. *Physiol. Mol. Plant Pathol.* 121:101859. 10.1016/J.PMPP.2022.101859

[B12] Fang W. Wang X. Huang B. Zhang D. Liu J. Zhu J. (2020). Comparative analysis of the effects of five soil fumigants on the abundance of denitrifying microbes and changes in bacterial community composition. *Ecotox. Environ. Safe.* 187:109850. 10.1016/j.ecoenv.2019.109850 31677569

[B13] Fernando P. C. (2006). Agriculture, pesticides, food security and food safety. *Environ. Sci. Policy* 9 685–692. 10.1016/j.envsci.2006.08.002

[B14] Franz S. Richard Ö Ellen K. Rosa M. (1996). *Methods in soil biology.* Berlin: Springer-Verlag.

[B15] Geng Y. Wang D. Yang W. (2017). Effects of different inundation periods on soil enzyme activity in riparian zones in Lijiang. *Catena* 149 19–27. 10.1016/j.catena.2016.08.004

[B16] Gwenaël I. Stéphane V. (2012). Measuring the effects of pesticides on bacterial communities in soil: A critical review. *Eur. J. Soil Biol.* 49 22–30. 10.1016/j.ejsobi.2011.11.010

[B17] Haas D. Défago G. (2005). Biological control of soil-borne pathogens by fluorescent pseudomonads. *Nat. Rev. Microbiol.* 3 307–319. 10.1038/nrmicro1129 15759041

[B18] Harrier L. A. Watson C. A. (2004). The potential role of arbuscular mycorrhizal (AM) fungi in the bioprotection of plants against soil-borne pathogens in organic and/or other sustainable farming systems. *Pest Manag. Sci.* 60 149–157. 10.1002/ps.820 14971681

[B19] Huang B. Yan D. Ouyang C. Zhang D. Zhu J. Liu J. (2020). Chloropicrin fumigation alters the soil phosphorus and the composition of the encoding alkaline phosphatase phod gene microbial community. *Sci. Total Environ.* 711:135080. 10.1016/j.scitotenv.2019.135080 31818557

[B20] Inmaculada S. Daniel P. Laura T. Esther P. César A. Inmaculada L. (2020). Effects of halophyte root exudates and their components on chemotaxis, biofilm formation and colonization of the halophilic bacterium *Halomonas anticariensis* FP35T. *Microorganisms* 8:575. 10.3390/microorganisms8040575 32316222 PMC7232322

[B21] Karki K. Grant J. Biscaia R. Petkar A. Hajihassani A. Coolong T. (2021). Evaluation of efficacy of Pic-clor 60 [choloropicrin pre-mixed with 1,3 dicholoropropene] and soil-applied fungicides to manage *Fusarium* wilt in watermelon. *Crop Prot.* 154:105894. 10.1016/J.CROPRO.2021.105894

[B22] Kolde R. (2015). *Pheatmap: Pretty heatmaps. R package. Version 1.0.8.* http://cran.r-project.org/web/packages/pheatmap/index.html.

[B23] Li Q. Zhang D. Cheng H. Song Z. Ren L. Hao B. (2021a). Chloropicrin alternated with dazomet improved the soil’s physicochemical properties, changed microbial communities and increased strawberry yield. *Ecotoxicol. Environ. Saf.* 220:112362. 10.1016/j.ecoenv.2021.112362 34087650

[B24] Li Q. Zhang D. Song Z. Ren L. Jin X. Fang W. (2022). Organic fertilizer activates soil beneficial microorganisms to promote strawberry growth and soil health after fumigation. *Environ. Pollut.* 295:118653. 10.1016/J.ENVPOL.2021.118653 34921948

[B25] Li Z. Bai X. Jiao S. Li Y. Li P. Yang Y. (2021b). A simplified synthetic community rescues *Astragalus mongholicus* from root rot disease by activating plant-induced systemic resistance. *Microbiome* 9:217. 10.1186/S40168-021-01169-9 34732249 PMC8567675

[B26] Liang X. Jin Y. He M. Liu Y. Hua G. Wang S. (2017). Composition of phosphorus species and phosphatase activities in a paddy soil treated with manure at varying rates. *Agric. Ecosyst. Environ.* 237 173–180. 10.1016/j.agee.2016.12.033

[B27] Massi F. Torriani S. F. F. Borghi L. Toffolatti S. L. (2021). Fungicide resistance evolution and detection in plant pathogens: *Plasmopara viticola* as a case study. *Microorganisms* 9:119. 10.3390/microorganisms9010119 33419171 PMC7825580

[B28] Nan X. Tan G. Wang H. Gai X. (2016). Effect of biochar additions to soil on nitrogen leaching, microbial biomass and bacterial community structure. *Eur. J. Soil Biol.* 74 1–8. 10.1016/j.ejsobi.2016.02.004

[B29] National Microbial Rescoure Center (2013). *Microascus.* Pune, MH: National Microbial Rescoure Center.

[B30] Osorio N. W. Mitiku H. (2014). Soil phosphate desorption induced by a phosphate-solubilizing fungus. *Commun. Soil Sci. Plant Anal.* 45 451–460. 10.1080/00103624.2013.870190

[B31] Olsen S. (1954). *Estimation of available phosphorus in soils by extraction with sodium bicarbonate.* Washington, DC: US Department of Agriculture.

[B32] Oshiki M. Toyama Y. Suenaga T. Terada A. Kasahara Y. Yamaguchi T. (2022). N2O reduction by *Gemmatimonas aurantiaca* and potential involvement of Gemmatimonadetes bacteria in N2O reduction in agricultural soils. *Microbes Environ.* 37:ME21090. 10.1264/jsme2.ME21090 35418546 PMC9530729

[B33] Page A. L. (1983). *Methods of soil analysis: Part 2 chemical and microbiological properties.* Michigan: American Society of Agronomy, Soil Science Society of America.

[B34] Piotr S. Beata M. Hanna B. Eligio M. (2014). Changes in soil microbial populations after fumigation and alternative methods to control soil-borne diseases. *Plant Sci.* 51 79–81.

[B35] Rachel I. A. Marzia M. John W. T. Thomas D. B. (2013). Dispersal in microbes: Fungi in indoor air are dominated by outdoor air and show dispersal limitation at short distances. *ISME J.* 7 1460–1460. 10.1038/ismej.2013.84 PMC3695294 23426013

[B36] Roeland L. B. Corné M. J. P. Peter A. H. M. B. (2012). The rhizosphere microbiome and plant health. *Trends Plant Sci.* 17 478–486. 10.1016/j.tplants.2012.04.001 22564542

[B37] Schloss P. D. Westcott S. L. Ryabin T. Hall J. R. Hartmann M. Hollister E. B. (2009). Introducing mothur: Open-source, platform-independent, community-supported software for describing and comparing microbial communities. *Appl. Environ. Microbiol.* 75 7537–7541. 10.1128/AEM.01541-09 19801464 PMC2786419

[B38] Segata N. Izard J. Waldron L. Gevers D. Miropolsky L. Garrett W. S. (2011). Metagenomic biomarker discovery and explanation. *Genome Biol.* 12:R60. 10.1186/gb-2011-12-6-r60 21702898 PMC3218848

[B39] Singh D. K. Kumar S. (2008). Nitrate reductase, arginine deaminase, urease and dehydrogenase activities in natural soil (ridges with forest) and in cotton soil after acetamiprid treatments. *Chemosphere* 71 412–418. 10.1016/j.chemosphere.2007.11.005 18082867

[B40] Sohail K. Subhan F. Haleem K. S. Khattak M. N. K. Khan I. Sultan T. (2018). Impact of plant growth-promoting rhizobacteria on yield and disease control of *Nicotiana tabacum*. *Arch. Biol. Sci.* 70 717–725. 10.2298/ABS180315035K

[B41] Swify S. Avizienyte D. Mazeika R. Braziene Z. (2022). Influence of modified urea compounds to improve nitrogen use efficiency under corn growth system. *Sustainability* 14:14166. 10.3390/SU142114166

[B42] Wang Q. Garrity G. M. Tiedje J. M. Cole J. R. (2007). Naïve bayesian classifier for rapid assignment of rrna sequences into the new bacterial taxonomy. *Appl. Environ. Microbiol.* 73 5261–5267. 10.1128/AEM.00062-07 17586664 PMC1950982

[B43] Wu X. Li H. Wang Y. Zhang X. (2020). Effects of bio-organic fertiliser fortified by bacillus cereus qj-1 on tobacco bacterial wilt control and soil quality improvement. *Biocontrol Sci. Technol.* 30 351–369. 10.1080/09583157.2020.1711870

[B44] Yan D. Wang Q. Li Y. Guo M. Guo X. Ouyang C. (2021). Efficacy and economics evaluation of seed rhizome treatment combined with preplant soil fumigation on ginger soilborne disease, plant growth and yield promotion. *J. Sci. Food Agric.* 102 1894–1902. 10.1002/JSFA.11526 34510449

[B45] You L. Gong Y. Li L. Hu X. Charles B. Viktoryia K. (2020). Beneficial effects of three brown seaweed polysaccharides on gut microbiota and their structural characteristics: An overview. *Int. J. Food Sci. Technol.* 55 1196–1206. 10.1111/ijfs.14408

[B46] Zhang D. Cheng H. Hao B. Li Q. Wu J. Zhang Y. (2021). Fresh chicken manure fumigation reduces the inhibition time of chloropicrin on soil bacteria and fungi and increases beneficial microorganisms. *Environ. Pollut.* 286:117460. 10.1016/J.ENVPOL.2021.117460 34438480

[B47] Zhang L. Liu H. Dai J. Xu P. Tang H. (2022). Unveiling degradation mechanism of PAHs by a *Sphingobium* strain from a microbial consortium. *mLife* 1 287–302. 10.1002/MLF2.12032 PMC10989954 38818225

[B48] Zhang Z. Wang X. Han S. Liu C. Liu F. (2018). Effect of two seaweed polysaccharides on intestinal microbiota in mice evaluated by illumina PE250 sequencing. *Int. J. Biol. Macromol.* 112 796–802. 10.1016/j.ijbiomac.2018.01.192 29427682

[B49] Zhu J. Ren Z. Huang B. Cao A. Wang Q. Yan D. (2020). Effects of fumigation with allyl isothiocyanate on soil microbial diversity and community structure of tomato. *J. Agric. Food Chem.* 68 1226–1236. 10.1021/acs.jafc.9b07292 31922739

