# Supplementary material for: Application of microbial organic fertilizers promotes the utilization of nutrients and restoration of microbial community structure and function in rhizosphere soils after dazomet fumigation
Source: Front Microbiol. 2023 Jan 18;13:1122611. doi: 10.3389/fmicb.2022.1122611 (PMC9891460; doi:10.3389/fmicb.2022.1122611)
Supplement: Supplementary file 1 [file Table_1.DOCX]

**Table S1: Functional Relative abundance of fungal soil**

| Treatment | CK | DZ | | DZF1 | | DZF2 |  |
| --- | --- | --- | --- | --- | --- | --- | --- |
| Undefined Saprotroph  Plant Pathogen  Animal Pathogen-Endophyte-Lichen Parasite-Plant Pathogen-Soil Saprotroph-Wood Saprotroph  Animal Pathogen-Dung Saprotroph-Endophyte-Epiphyte-Plant Saprotroph-Wood Saprotroph  Unknown  Orchid Mycorrhizal-Plant Pathogen-Wood Saprotroph  Endophyte-Litter Saprotroph-Soil Saprotroph-Undefined Saprotroph  Animal Pathogen-Endophyte-Plant Pathogen-Wood Saprotroph  Wood Saprotroph  Animal Pathogen-Soil Saprotroph  Animal Parasite-Fungal Parasite  Animal Pathogen-Endophyte-Plant Pathogen-Undefined Saprotroph  Others | 20.33±1.91b  27.32±1.25ab  16.83±1.54a  0.08±0.02c  19.73±1.33a  0.01±0.00b  2.42±0.52a  1.09±0.50a  1.23±0.27a  5.37±2.47a  0.00±0.00a  0.00±0.00c  5.59±0.65ab | 13.46±0.75c  35.92±1.38a  4.96±1.31c  32.57±0.39a  7.88±0.47b  0.43±0.15b  0.44±0.07b  0.18±0.04a  0.10±0.03b  0.10±0.03b  0.14±0.03a  0.34±0.07b  3.48±0.71b | 17.83±1.33bc  20.55±2.72b  9.69±0.58b  28.22±9.63a  10.16±2.52b  0.07±0.04b  1.77±0.77ab  0.21±0.05a  0.28±0.07b  0.59±0.08b  2.58±2.53a  2.09±0.17a  5.96±0.51a | | 33.24±2.90a  31.92±6.42ab  11.05±1.65b  6.37±2.08b  6.01±0.13b  2.27±0.43a  1.17±0.28ab  0.73±0.21a  0.56±0.13b  0.38±0.10b  0.03±0.02a  0.02±0.00c  6.25±0.74a | | |

Note: CK: no treatment; DZ: fumigation with 300 kg hm^−1^ dazomet; DZF1: 300 kg hm^−1^ dazomet fumigation followed by 1200 kg hm^−1^ Junweinong MOF; DZF2: 300 kg hm^−1^ dazomet fumigation followed by 1200 kg hm^−1^ Junlisu MOF. Means (N=3) within the same column accompanied by the same letter following by Duncan's new multiple range test are not statistically different (p= 0.05. a, b and ab are significant markers, different letters indicate significant differences, as long as one letter is the same, it is not significant).
